# Determinants of high vaccine confidence and uptake among the Australian public: insights from a cross-sectional study

**DOI:** 10.3389/fpubh.2025.1513892

**Published:** 2025-05-30

**Authors:** Charles Travers Williams, Bandana Saini, Syed Tabish R. Zaidi, Ronald L. Castelino

**Affiliations:** ^1^Faculty of Medicine and Health, University of Sydney, Camperdown, NSW, Australia; ^2^Laborator School of Pharmacy and Pharmacology, College of Medicine and Health, University of Tasmania, Hobart, TAS, Australia; ^3^Pharmacy Department, Blacktown Hospital, Blacktown, NSW, Australia

**Keywords:** vaccine hesitancy, vaccines, pandemic, immunization, Australia, COVID-19

## Abstract

**Background:**

In 2021, Australia launched a national COVID-19 vaccine rollout strategy, but encountered setbacks that resulted in negative press and media controversy. This study aimed to confirm factors influencing vaccine confidence and uptake in Australian adults and validate the use of a Vaccine Confidence Scale.

**Methods:**

A cross-sectional study was conducted from 15 to 21 September 2021, coinciding with the expansion of COVID-19 BNT162b2 vaccine eligibility to all adults in Australia. A voluntary online survey assessed vaccine confidence and influencing factors among adults (≥18 years of age). Multivariate logistic regression was used to identify predictors of high vaccination confidence (score >30) and uptake using odds ratios (OR) with 95% confidence intervals to measure effect size.

**Results:**

Among 471 respondents, vaccine confidence (mean score 20/40) and uptake (29.7%) was low. Those who used government websites (OR 6.35; *p* < 0.001) and general practitioners (OR 4.05; *p* < 0.001), as sources of COVID-19 information, or had received a healthcare professional recommendation (OR 2.82; *p* < 0.01) were up to six times more likely to have high vaccine confidence, and were more likely to receive a vaccination. In contrast, the use of non-mainstream media and online sources decreased confidence and reduced the likelihood of vaccination by ~60% (OR 0.37; *p* < 0.05). The Vaccine Confidence Scale demonstrated potential as a tool for rapidly assessing vaccine confidence and predicting the likelihood of vaccine uptake.

**Conclusion:**

Greater emphasis should be placed on raising awareness of trusted sources for vaccine information, and immunization programs should consider incorporating vaccine confidence tools to enhance communication strategies and encourage vaccine uptake.

## 1 Introduction

The COVID-19 pandemic plunged the world into an unprecedented health crisis, disrupting societies and straining healthcare systems globally. Australia's first encounter with this disease began with the confirmation of its first COVID-19 case in Victoria on January 25, 2020 (1). Since then, the country reported over 11 million cases and mourned more than 23,000 lives lost (2), underscoring the profound public health toll of this disease.

As COVID-19 transitions from a pandemic to an endemic phase, the insights gained from this period continue to shape public health interventions and vaccination programmes. In response to the urgency of the situation, novel COVID-19 vaccines were developed and swiftly rolled by the Australian government in January 2021 under the COVID-19 Vaccine National Roll-out Strategy, prioritizing distribution based upon population group vulnerability (3, 4). However, the rollout encountered several setbacks that fuelled negative narratives and misinformation in the traditional press and social media spheres, impacting initial COVID-19 vaccine uptake (4, 5).

In April 2021, safety concerns surrounding the government-chosen ChAdOx1-S vaccine were raised, particularly regarding a rare but serious side effect known as thrombosis with thrombocytopenia (TTS) which had higher reported rates in younger adults (6). Due to a higher risk of TTS with the ChAdOx1-S vaccine in younger adults, the BNT162b2 vaccine was instead recommended first for those under 50 years of age (4, 6). However, subsequent revisions in June 2021 increased the age threshold to 60 years, as further evidence emerged of heightened TTS risk among adults aged 50–59 years. Yet, the persistent challenge of limited BNT162b2 supply meant that healthy adults under 40 years remained unable to receive the vaccine until August 2021 in most regions of Australia. This led to negative media coverage and the spread of vaccine misinformation, and this social amplification of vaccine-related risks, may have eroded COVID-19 vaccine confidence among the general public (7).

Vaccine confidence refers to the belief in the effectiveness and safety of vaccines and trust in the systems that deliver them, ranging from no confidence to complete confidence (8). While distinct from vaccine hesitancy (the motivational state of being conflicted about or opposed to getting vaccinated) (8), low vaccine confidence can contribute to hesitancy, impacting the decision to get vaccinated and overall vaccine uptake. Introduced in the early 21st century (9, 10), vaccine confidence emerged in response to growing hesitancy driven by misinformation, such as the debunked link between the measles, mumps, and rubella vaccine and autism (11). The Vaccine Confidence Project (VCP) established in 2010, along with the WHO's Strategic Advisory Group of Experts (SAGE), has been instrumental in studying and mapping vaccine hesitancy globally, identifying key factors such as “convenience,” “complacency,” and “confidence” as reasons for hesitancy (10). The 2022 EU VCP report indicated a decline in vaccine confidence during the COVID-19 pandemic, underscoring the need to understand these trends for future vaccination strategies (12). High vaccine confidence is essential for achieving high coverage and herd immunity, reducing disease transmission, and protecting those who cannot be vaccinated (13). On the contrary, low confidence can lead to outbreaks and prolonged public health crises, making it crucial for public health bodies to foster vaccine confidence to safeguard public health (14).

A recently published single-center, cross-sectional study in Australia conducted following the vaccine advice changes highlighted that vaccine confidence was high in adults who had already chosen to receive a COVID-19 vaccination and demonstrated that an individual's source of information was a key influencing factor (15). In this research, recruitment occurred in a hospital setting by inviting adults to complete a digital survey during the observation period following their vaccination. An eight-item, three-factor measure of vaccination confidence was found to be a reliable way to measure vaccination beliefs among these COVID-19-vaccinated individuals. However, whether this holds true for the general population who may not have yet received a COVID-19 vaccine remains unknown.

The aim of the current study, therefore was to assess vaccine confidence in a general sample of Australian adults (≥18 years of age) following the vaccine rollout changes and confirm the factors influencing vaccine confidence and uptake.

## 2 Materials and methods

### 2.1 Study design and data collection

This cross-sectional study was carried out from 15 to 21 September 2021, coinciding with the expansion of COVID-19 BNT162b2 vaccine eligibility to all adults in Australia (16).

Adults ≥18 years of age in Australia were invited via Facebook to share their views on COVID-19 vaccines and the pandemic by completing a 10-min online anonymised survey. The Research Electronic Data Capture (REDCap) tool, a secure web-based database application, was used to conduct the survey, and completion was considered as implied consent.

The Human Research Ethics Committee and Western Sydney Local Health District granted approval to conduct this study (2021/ETH01038/STE02184).

### 2.2 Measures

The details of the survey development have been previously reported in a preliminary study by Williams et al. (15). The survey utilized in this second study was identical in nature with the only exception being that the survey in this study did not assume that participants had already received a COVID-19 vaccination and additionally assessed their COVID-19 vaccination status and whether they were healthcare professionals (see Supplementary material).

In summary, a modified 24-item, two-part survey was used to assess vaccine confidence and influencing factors. Part 1 (16 items) evaluated factors associated with vaccine confidence and uptake, adapted from the SAGE Working Group on Vaccine Hesitancy and the CDC's COVID-19 Vaccine Confidence: Rapid Community Assessment Tool (17, 18). It covered four domains: Conditional (demographic, socioeconomic), Social (news and COVID-19 information sources), Motivation (vaccination drivers), and Practical influences (ease of access). Part 2 measured COVID-19 vaccine confidence with an 8-item scale adapted from a validated Vaccine Confidence Scale for parents of adolescents, using the Health Belief Model (19). Responses on a 5-point Likert scale assessed perceived Benefits, perceived Harm, and Trust domains. Total confidence scores ranged from 8 to 40, categorized into low ( ≤ 20), medium (21–30), and high (>30) confidence.

### 2.3 Data analysis

Descriptive statistics summarized demographic characteristics, vaccine confidence, and the frequency of conditional, social, motivational, and practical influences. Chi-square tests analyzed differences in demographics and vaccination motivators for discrete and non-normally distributed data, while t-tests were used for continuous and normally distributed data. Univariate and multivariate logistic regression analyzed the relationship between high COVID-19 vaccination confidence and potential predictors for complete data, using odds ratios with 95% confidence intervals (CI) to measure effect size. Variables with significant univariate associations were included in the multivariable model. Confirmatory factor analysis assessed the adapted 8-item Vaccine Confidence Scale's construct validity and fit using the preferred three-factor model (“Benefits,” “Harm,” “Trust”) identified previously in the preliminary study by Williams et al. (15). Model goodness of fit was evaluated with the comparative fit index (CFI) and root mean square of approximation (RMSEA), with acceptable fit defined as CFI >0.90 and RMSEA < 0.08 (20, 21). A chi-squared goodness-of-fit test checked model distribution uniformity. Logistic regression was used to assess the association between vaccine confidence and vaccination status (i.e., scale predictive validity). Cronbach's alpha coefficients assessed scale reliability, with α ≥0.7 indicating acceptable reliability (22). Statistical analyses were performed using IBM SPSS (version 29.0), with significance set at *p* < 0.05.

## 3 Results

### 3.1 Sociodemographic and economic characteristics

Overall, 471 respondents completed the survey and were included in the final analysis (Table 1). Of those, 359 had complete data (76.2%). There was a similar proportion of male and female respondents (47.9% and 46.7%, respectively). Most respondents were 55–64 years of age (28.2%), followed by 45–54 (24.4%), 65–74 (22.3%), and 35–44 (11.5%) years of age. The majority identified themselves as being of Australian ancestry (68.6%) with Christianity (48.2%) and atheism (28.2%) as the most commonly reported religious beliefs. The most common highest level of education among respondents was high school (33.8%). The distribution of reported household income was positively skewed with most respondents on a weekly income ≤ $1,500 AUD per week (38.6%). A large portion of respondents (42.6%) had a medical condition or risk factor associated with a high risk of severe COVID-19; the most frequently reported being asthma (16.1%), hypertension (14.6%), and current smoking status (9.6%). Approximately one-fifth (21.2%) had experienced COVID-19 disease either personally or had a family member or friend who had the disease. A COVID-19 vaccine recommendation was provided by a HCP to 32.1% of the respondents and 29.7% reported having received a COVID-19 vaccination.

**Table 1:** Respondent demographics.

***N* = 471**	***n***	**%**
**Gender**		
Female	220	46.7
Male	226	47.9
Other/non-binary	21	4.5
**Age**		
18–24	8	1.7
25–34	22	4.7
35–44	54	11.5
45–54	115	24.4
55–64	133	28.2
65–74	105	22.3
75+	22	4.7
**Ancestry**		
Aboriginal/Torres Strait Islander	8	1.7
Australian	323	68.6
Dutch	100	21.2
English	17	3.6
German	15	3.2
Irish	17	3.6
Italian	15	3.2
Scottish	25	5.3
Other ancestry^*^	43	9.1
**Religion**		
Buddhism	6	1.3
Christian	227	48.2
Islam	2	8.2
No religion	113	28.2
Other religion^†^	20	4.2
**Education**		
Less than high school	9	1.9
High school	159	33.8
Bachelor's degree	112	24.0
Master's degree	39	8.3
PhD or higher	17	3.6
Trade school	72	15.3
**Is a self-reported HCP**		
Yes	62	13.2
**Income**		
$1–500 per week	57	12.1
$501–1,000 per week	67	14.2
$1,001–1,500 per week	58	12.3
$1,501–2,000 per week	34	7.2
$2,001–2,500 per week	33	7.0
>$2,500 per week	49	10.4
**Medical condition assoc. high risk of severe COVID-19** ^‡^		
Yes	200	42.6
**Previous COVID-19 experience** ^§^		
Yes	100	21.2
**HCP recommended the vaccine**		
Yes	151	32.1
**Have received a COVID-19 vaccination**		
Yes	140	29.7

^*^Most common other ancestries were South African (0.8%), Croatian (0.6%), Greek (0.6%), and Polish (0.6%).

^†^Most common other religions were Agnostic (0.4%) and Judaism (0.2%).

^‡^Most common conditions were asthma (16.1%), hypertension (14.6%), smoker (9.6%), obesity (8.1%), diabetes (7.9%), and heart disease (7.0%).

^§^The respondent or someone in their family or friends had COVID-19.

### 3.2 Sources of news and COVID-19 information

**Figure 1:**
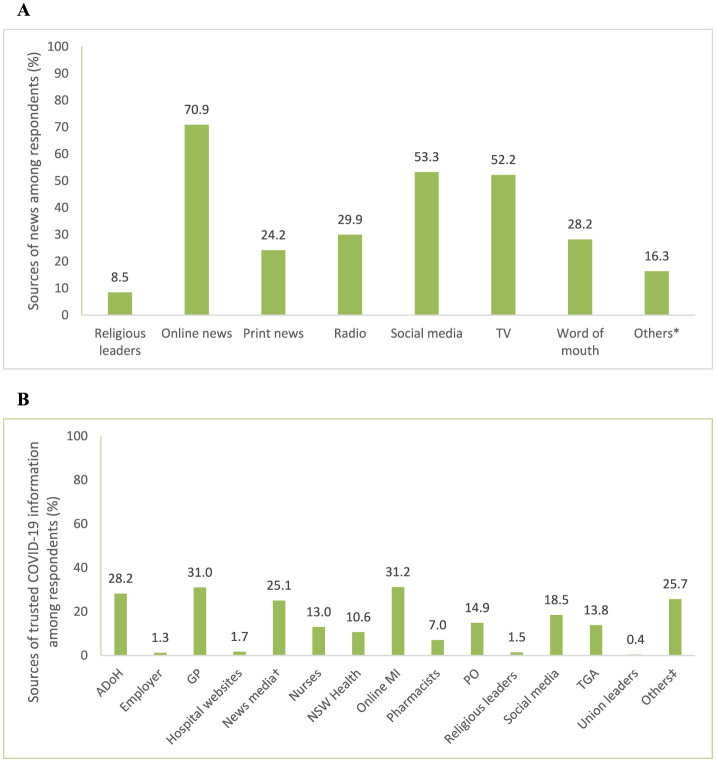
**(A)** Sources of news and **(B)** trusted sources of COVID-19 information reported among respondents. *Other sources of news included podcasts, YouTube, and government websites. ^†^Television, internet, radio, ^‡^Other trusted sources of COVID-19 information included non-mainstream media/online sources, personal research/experiences, friends and family. ADoH, Australian Department of Health; CDC, Centers for Disease Control and Prevention; GP, general practitioner; MI, medical information; NSW, New South Wales; PO, professional organizations; TGA, therapeutic goods administration; WHO, World Health Organization.

The sources from which respondents reported getting their news and trusted COVID-19 information are shown in Figures 1A, B, respectively. The most frequently cited sources of news were online news articles (*n* = 334; 70.9%), social media (*n* = 251; 53.3%), and TV (*n* = 246; 52.2%). For COVID-19 information, the three most trusted sources were independent online medical information (e.g., WebMD; *n* = 147; 31.2%), general practitioners (*n* = 146; 31.0%), and the Australian Department of Health (ADoH; *n* = 133; 28.2%). The news media (*n* = 118; 25.1%), other sources of COVID-19 information (*n* = 121; 25.7%), and social media (*n* = 87; 18.5%) followed close behind. Other sources of COVID-19 information included non-mainstream media/online sources (e.g., forums; *n* = 14; 3.0%), personal research/experiences (*n* = 12; 2.5%), and friends and family (*n* = 9; 1.9%). When asked if they had encountered any information about COVID-19 vaccines they could not determine were true or false, more than three-quarters of respondents (*n* = 364; 77.3%) indicated they might have been exposed to “fake news” or misinformation.

### 3.3. Motivators for receiving a vaccination

**Figure 2:**
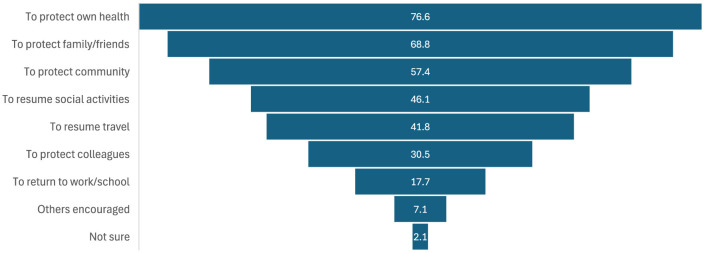
Reported motivators for receiving a COVID-19 vaccination (%).

For the respondents who received a COVID-19 vaccine (*n* = 141), the reported motivators for getting vaccinated are shown in Figure 2. The most frequently cited motivator was protecting one's own health (76.6%; *n* = 108), followed by protecting family and friends (68.8%; *n* = 97), and protecting the community (57.4%; *n* = 81). Female respondents were more likely to be motivated to get vaccinated by the notion of protecting their community (*X*^2^ = 6.23; *p* = 0.013). While respondents < 65 years of age were more likely to be motivated to get back to work or school (*X*^2^ = 11.90; *p* < 0.001) and to protect the health of their colleagues (*X*^2^ = 7.00; *p* = 0.008).

### 3.4 Predictors of high vaccine confidence

Overall vaccine confidence scores were low, with a mean score of 20.0 (SD 9.34). Among the respondents, 62.8% (*n* = 296) were categorized as having low vaccine confidence, 17.4% (*n* = 82) as medium, and 19.7% (*n* = 93) as high. Most respondents did not agree with the perceived benefits of COVID-19 vaccines or the perceived harms of contracting COVID-19 (see Supplementary material, Supplementary Figures S1–S3). Although there was a general trust in GPs, the majority of respondents were skeptical about the government's intentions regarding COVID-19 vaccinations. When comparing mean scores across the perceived Benefits, Harm, and Trust domains (possible range of 1–5 for each), respondents generally rated Benefits [four items, mean 2.32; standard error (SE) 0.06] lower than Harm (two items, mean 2.52; SE 0.06) and Trust (two items, mean 2.86; SE 0.05).

Initial univariate analysis identified 15 predictors significantly associated with high vaccine confidence (total score >30; see Table 2). Following multivariate analysis, five predictors remained significant. The strongest predictor of high vaccine confidence was the source of COVID-19 information. Use of the ADoH (OR 6.17; *p* < 0.001), GPs (OR 4.05; *p* < 0.001), and state health websites (OR 6.35; *p* < 0.001) were positively associated with high vaccine confidence. Conversely, reliance on other sources of COVID-19 information, such as non-mainstream media and online websites, was the strongest negative predictor (OR 0.20; *p* = 0.04). Lastly, respondents who received a recommendation from an HCP to get the COVID-19 vaccine were more likely to have high vaccine confidence (OR 2.82; *p* = 0.002).

**Table 2:** Predictors of high vaccine confidence (total score >30).

**Predictor variable**	**Univariate analysis**			**Multivariate analysis**		
	**OR**	**95% CI**	***p*** **-value**	**OR**	**95% CI**	***p*** **-value**
Age ≥65 years	2.15	1.33–3.47	0.002	0.94	0.46–1.92	0.872
Australian	1.87	1.09–3.20	0.023	0.82	0.37–1.83	0.633
COVID-19: ADoH	19.35	10.98–34.08	< 0.001	**6.17**	**3.10–12.30**	**< 0.001**
COVID-19: GP	7.97	4.82–13.20	< 0.001	**4.05**	**1.97–8.34**	**< 0.001**
COVID-19: state health	13.04	6.77–25.10	< 0.001	**6.35**	**2.57–15.65**	**< 0.001**
COVID-19: other sources^*^	0.05	0.01–0.20	< 0.001	**0.20**	**0.04–0.93**	**0.040**
COVID-19: social media	0.08	0.02–0.31	< 0.001	0.38	0.08–1.84	0.230
Exposure to fake news	0.59	0.35–0.99	0.044	0.79	0.37–1.71	0.555
Gender	1.95	1.22–3.12	0.005	1.61	0.82–3.17	0.168
HCP recommended	4.38	2.70–7.10	< 0.001	**2.82**	**1.44–5.53**	**0.002**
Irish	3.00	1.11–8.09	0.031	2.47	0.59–10.31	0.214
Medical condition	0.55	0.34–0.89	0.015	0.70	0.34–1.46	0.344
News: religious leaders	0.20	0.05–0.83	0.027	0.17	0.017–1.83	0.145
News: social media	0.51	0.32–0.80	0.004	0.54	0.26–1.12	0.092
News: word of mouth	0.55	0.31–0.96	0.035	1.08	0.44–2.66	0.869

ADoH, Australian Department of Health; CI, confidence interval; GP, general practitioner; HCP, healthcare professional; OR, odds ratio.

^*^Such as, non-mainstream media/online sources, personal research/experiences, friends and family. Bold indicates significance after adjusting for all covariates.

### 3.5 Association with vaccination status and scale validation

Vaccine confidence scores were positively associated with vaccination status with every one-point increase in total score corresponding to a 34% increase in the odds of COVID-19 vaccination (OR 1.34; 95% CI 1.27–1.40). Similarly, mean overall scale and subscale scores (Benefits, Harms, and Trust) were all significantly associated with vaccination (Supplementary Table S1), with the overall scale demonstrating the strongest association (OR 9.99; *p* < 0.001). Univariate and multivariate analyses also identified similar predictors associated with vaccination as for those for high vaccine confidence (Supplementary Table S2). Following multivariate analysis, positive predictors for receiving a COVID vaccination were the use of the ADoH (OR 4.36; *p* < 0.001), GPs (OR 4.57; *p* < 0.001), and state health websites (OR 8.62; *p* < 0.001) for vaccine information and receipt of an HCP recommendation (OR 1.87; *p* = 0.034). Whereas use of other sources of COVID-19 information was a negative predictor of vaccination (OR 0.37; *p* = 0.035).

When stratifying respondents by COVID-19 vaccination status, those who had already received a COVID-19 vaccination had higher mean overall vaccine confidence scores compared with those who had not [31.10 (SE 0.62) vs. 15.20 (SE 0.29); *t*-test, *p* < 0.001], demonstrating face validity of the Vaccine Confidence Scale. Mean subscale scores were also higher for COVID-19 vaccinated [Benefit = 3.99 (SE 0.09); Harm = 3.72 (SE 0.08); Trust = 3.85 (SE 0.08)] vs. unvaccinated [Benefit = 1.59 (SE 0.04); Harm = 1.99 (SE 0.06); Trust = 2.43 (SE 0.04)] respondents (*p* < 0.001 for all).

**Figure 3:**
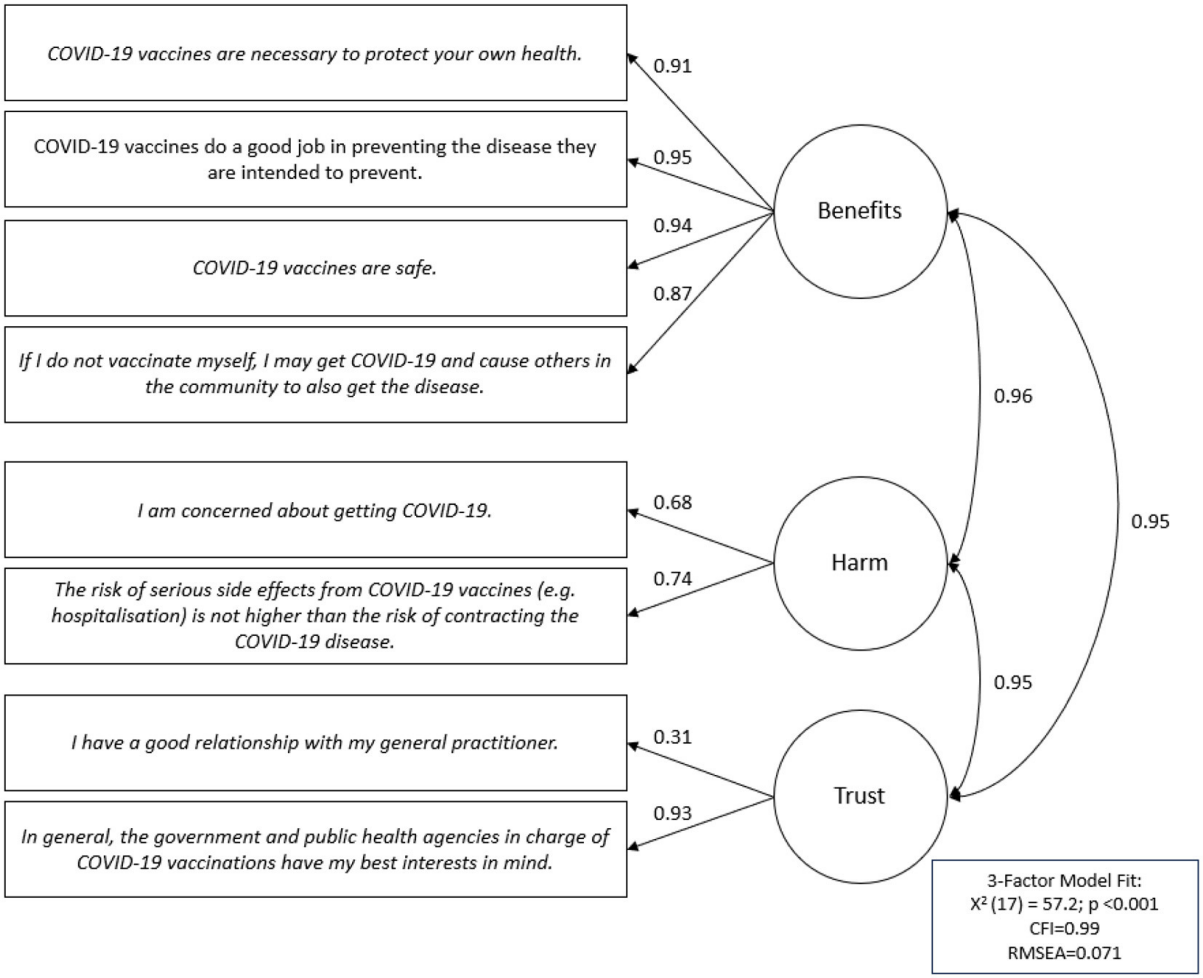
Confirmatory factor analysis of the Vaccine Confidence 3-factor scale with standardized factor loading values.

When assessing scale construct validity, the three-factor Vaccine Confidence Scale (Benefits, Harm, and Trust) showed good model fit with a CFI = 0.99, RMSEA = 0.071, and an *X*^2^(17) = 57.2 (*p* < 0.001; Figure 3). Standardized factor loadings for the Benefits, Harm, and Trust subscales ranged from 0.87–0.91, 0.68–0.74, and 0.31–0.93, respectively (Supplementary Table S3). Strong internal scale consistency was also demonstrated with an overall Cronbach's alpha coefficient of 0.93 (Benefits α = 0.95; Harm α = 0.67; Trust α = 0.44).

## 4 Discussion

In this study, factors influencing COVID-19 vaccine confidence and uptake in Australian adults (≥18 years of age) were assessed using an online cross-sectional survey following national vaccine rollout changes and negative media controversy. The study found that overall vaccine confidence was low and that less than one-third of respondents had received a COVID-19 vaccine. Use of government websites and HCPs, such as GPs, as sources of vaccine information, and receiving an HCP recommendation for vaccination resulted in higher vaccine confidence and likelihood of vaccine uptake. In contrast, exposure to other sources of vaccine information, such as non-mainstream media and online sources, appeared to undermine confidence and the likelihood of vaccination.

In our previous study at Blacktown Hospital, Sydney, Australia (4 August−14 September 2021; *N* = 1,053), respondents who had just received a COVID-19 vaccine had high vaccine confidence (mean total score of 33.0), which was to be expected (15). In contrast, this study conducted in the wider population and immediately after the previous study (15–21 September 2021), reported low vaccine confidence (mean total score of 20.0) and poor vaccine coverage (29.7%). This suggests that the vaccine roll-out changes in April and June 2021 and the associated negative media controversy that followed (4), indeed may have been detrimental to COVID-19 vaccine confidence among Australian adults. Multiple studies have demonstrated that media coverage can erode vaccine confidence and contribute to vaccine hesitancy. For instance, Catalan-Matamoros et al. identified a significant inverse correlation between negative newspaper coverage from 2012 to 2017 and childhood vaccination rates in Spain (*r* = −0.771; *p* < 0.05) (23). Similarly, Suppli et al. (24) reported a 36% decline in HPV vaccination uptake among girls born in Denmark in 2003 following a surge in negative media coverage. This study further supports the potential harmful effects of media on vaccine confidence and its consequences on vaccination rates.

When assessing predictors of high vaccine confidence (total score >30), similar to our previous study (15), the use of government websites as sources of vaccine information was identified as a positive predictor, confirming the importance of utilizing trusted sources, such as government health bodies and HCPs, to disseminate vaccine information. Respondents in our current study who used these sources to access vaccine information were up to six times more likely to have high vaccine confidence and up to eight times more likely to receive a COVID-19 vaccination (Table 2 and Supplementary Table S2, respectively). Furthermore, respondents who were recommended by an HCP to receive a COVID-19 vaccine were almost three and two times more likely to have high vaccine confidence and receive a vaccination, respectively. Similar findings were reported in a recently published UK cross-sectional survey study (16–31 July 2021; *N* = 4,428) which highlighted that the use of NHS and government websites, and GPs for COVID-19 information were associated with a positive vaccination status (25). The sharing of vaccine information via trusted sources, such as government and health agencies or non-profit organizations, is essential, particularly during disease outbreaks. However, a study of COVID-19 vaccination websites from 58 countries found that only two met the recommended readability level for public materials (26). Similarly, in another study of 23 government websites, just 65% of these sites provided specific communication channels for COVID-19-related inquiries (27), underscoring the need to enhance the effectiveness and frequency of government communications on healthcare and vaccination.

Equally important is the exposure to untrusted information sources and the impact of misinformation. In this study, the use of non-mainstream media and online sources negatively impacted vaccine confidence and reduced the likelihood of COVID-19 vaccination by ~60%. Similarly, self-reported exposure to fake news was found to be a negative predictor of high vaccine confidence and vaccine uptake on univariate analyses. However, this was no longer significant following multivariate analyses likely due to the high proportion of respondents (77.3%) reporting exposure to fake news and the survey being conducted on a social media platform where misinformation is rampant. In our previous study at Blacktown Hospital, fewer respondents reported being exposed to fake news or misinformation (57.5%) but exposure appeared to decrease the likelihood of high vaccine confidence by almost 30% (OR 0.71; 95% CI 0.52–0.96) (15). Similarly, in a randomized controlled trial in the UK and USA (7–14 September 2020; *N* = 8,001), recent exposure to misinformation reduced the intent to accept a COVID-19 vaccine by 6.2 and 6.4 percentage points, respectively (28). Misinformation that used scientific messaging and imagery was found to be more strongly associated with declines in vaccination intent. Emerging research on theory-informed debiasing interventions, such as debunking, show promise in combating misinformation (29). These interventions should be tailored specifically to address vaccine misinformation, as this research is crucial for managing future pandemics.

For those respondents who had received a COVID-19 vaccination, the top three key reported motivators were to protect one's own health, family/friends, and/or the community (57.4%−76.6%), and were identical to our previous study at Blacktown Hospital (15). Interestingly, female respondents were more likely to be motivated to get vaccinated by the notion of altruistic notions of protecting others, while working adults (< 65 years of age) were more motivated by the prospect of returning to work and protecting their colleagues. While the notion of workers wanting to return to their routine life following lockdowns seems more obvious, gender differences in vaccine motivations are less so. A 2021 systematic review by Zintel et al. (30) found that males were more likely to be motivated than females to receive a COVID-19 vaccine and this difference was greater among healthcare workers compared with the general population. Although our current study did not specifically seek to explore gender differences, *post-hoc* analyses did indeed reveal differences in mean vaccine confidence levels and vaccination rates between males and females (*p* < 0.05 for both, results not presented). Potential gender discrepancies in vaccine confidence and motivators for uptake should be considered when implementing immunization programmes and further studies specifically designed to explore this concept are warranted.

This study made use of an eight-item scale to assess vaccine confidence and offered an efficient measure of adult vaccination beliefs. The scale, adapted from Gilkey et al.'s (19, 31) scale developed in parents of adolescents, and initially tested in our previous study at Blacktown Hospital (15), was further validated in this study of adults from the general population. Predictive validity was demonstrated with a one-point increase in total vaccine confidence score associated with a 34% increase in the likelihood of COVID-19 vaccination and multivariable analyses revealed identical positive and negative predictors for vaccination status as for high vaccine confidence. Face validity tests further highlighted that those who had already received a COVID-19 vaccination did indeed have higher overall mean vaccine confidence and subscale scores than adults who had not received a vaccination. The vaccine confidence scale also demonstrated good construct and internal consistency, with three-factor scale confirmatory factor analysis results revealing good model fit (CFI = 0.99; RMSEA = 0.071; X^2^(17) = 57.2, p < 0.001) and acceptable reliability (α = 0.93). The results were similar to our previous study at Blacktown Hospital of adults that had already received a COVID-19 vaccination, which also showed good model fit [CFI = 0.97; RMSEA = 0.071; *X*^2^(17) = 105.9, *p* < 0.001] and internal consistency (α = 0.82) with the adapted Vaccine Confidence Scale (15).

Several scales have been developed to measure vaccine confidence, and most highlight similar findings: perceived benefits of vaccines are strong predictors of vaccination behaviors (15, 32, 33). Some scales, like those used by Kranzler et al. (32) and MacEwan et al. (33), found that the benefits, such as community protection and minimal side effects, as key predictors. Others, like Luyten et al.'s (34), suggest that risk aversion may also influence vaccine decisions. However, in our study, trust (OR 8.36; *p* < 0.001) emerged as an especially significant predictor, surpassing perceived benefits and harms (OR 6.59; *p* < 0.001 and OR 4.36; *p* < 0.001, respectively). This indicates that, while vaccine campaigns should focus on promoting vaccine benefits and disease risks, they must also come from trusted sources to effectively impact vaccine confidence.

Although this study assessed determinants of high confidence and uptake for COVID-19 vaccines, the results may also apply to other adult vaccines, such as influenza. A recent US cross-sectional survey study that compared predictors of influenza (*n* = 1,136) and COVID-19 (*n* = 1,131) vaccine confidence, found that positive attitudes toward vaccination for both were driven by perceived virus severity, vaccine efficacy and adverse effects (35). Similarly, misinformation was negatively associated with the attitudes toward both vaccines. However, some differences are likely present and future research exploring these nuances could provide a deeper understanding of vaccine confidence and uptake drivers across different vaccines.

To our knowledge, this study is the first to cross-validate an adult Vaccine Confidence Scale using COVID-19 vaccines across different settings and by vaccination status (15). Although the study contributes valuable insights into the factors associated with high vaccine confidence and the motivations for receiving a vaccination, several limitations should be noted. (1) As a cross-sectional and observational study, it cannot establish causal relationships. Longitudinal data are needed to determine if predictors of high vaccine confidence persist and if they apply to booster doses of vaccines. (2) The study was conducted in Australia only, which may limit the generalizability of the findings. (3) Only Facebook was used to distribute the survey. Although it remains the most widely used social media platform in Australia, as not all individuals use Facebook there is a potential for sampling bias. However, several peer-reviewed studies show that surveys administered via Facebook have minimal bias compared with traditional surveys (36–38). (4) Although all analyses were conducted with complete data, item nonresponses (24.8%) may have led to non-response bias. (5) The type of COVID-19 vaccine received was not assessed, which could influence vaccine confidence, although access to alternative vaccines was limited at the time, with most participants likely receiving the BNT162b2 vaccine. Additionally, the study did not capture the history of other previously received vaccines, potentially leading to residual confounding. (6) Similarly, the state where respondents were from was not captured and state differences in vaccine confidence may exist, depending on state-provided health and vaccine information methods. Further research into regional and demographic differences in vaccine confidence is warranted and may help to support the development of more effective public health interventions with tailored communication strategies to support vaccine uptake.

## 5 Conclusion

In September 2021, COVID-19 vaccine confidence was low among adults in Australia likely due to the changes to the national vaccine roll-out strategy and surrounding negative media at the time. Individuals who received vaccine information from government health sites were up to six times more likely to have high vaccine confidence and up to eight times more likely to receive a COVID-19 vaccination. While the use of non-mainstream media and online sources undermined vaccine confidence and reduced the likelihood of vaccination by around 60%. The Vaccine Confidence Scale validated in this study shows promise as a tool for quickly assessing vaccine confidence and predicting the likelihood of vaccine uptake. Further efforts should be placed on increasing the awareness of trusted sources of vaccine information and public health interventions and immunization programmes should consider the use of vaccine confidence tools to optimize communication strategies and support vaccine uptake.

## Data Availability

The original contributions presented in the study are included in the article/Supplementary material, further inquiries can be directed to the corresponding authors.
