# Supplementary material for: Determinants of high vaccine confidence and uptake among the Australian public: insights from a cross-sectional study
Source: Front Public Health. 2025 May 30;13:1513892. doi: 10.3389/fpubh.2025.1513892 (PMC12162927; doi:10.3389/fpubh.2025.1513892)
Supplement: Supplementary file 1 [file Data_Sheet_1.docx]

Supplementary Material

# Supplementary Figures and Tables

## Figure S1. Perceived benefits: distribution of Likert score results from respondents agreeing or disagreeing with the four statements pertaining to the benefits of COVID-19 vaccination

## Figure S2. Perceived harm: distribution of Likert score results from respondents agreeing or disagreeing with the two statements pertaining to the harm of COVID-19 disease

## Figure S3. Perceived trust: distribution of Likert score results from respondents agreeing or disagreeing with the two statements pertaining to the trust of healthcare systems and government agencies

## Table S1. Scale and subscale mean vaccine confidence scores and univariable association with vaccination status

|  | **Vx**  **status, n (%)** | **Overall (8 items)** | | | **Benefits (4 items)** | | | **Harms (2 items)** | | | **Trust (2 items)** | | |
| --- | --- | --- | --- | --- | --- | --- | --- | --- | --- | --- | --- | --- | --- |
|  |  | **Mean (SE)** | **OR**  **(95% CI)** | **p-value** | **Mean (SE)** | **OR**  **(95% CI)** | **p-value** | **Mean (SE)** | **OR**  **(95% CI)** | **p-value** | **Mean (SE)** | **OR**  **(95% CI)** | **p-value** |
| No | 302 (64.1) | 1.90 (0.04) | Reference | | 1.59 (0.04) | Reference | | 1.99 (0.06) | Reference | | 2.43 (0.04) | Reference | |
| Yes | 140 (29.7) | 3.89 (0.08) | 9.99  (6.68–14.96) | <0.001 | 3.99 (0.09) | 6.59  (4.76–9.12) | <0.001 | 3.72 (0.08) | 4.36  (3.31–5.74) | <0.001 | 3.85 (0.08) | 8.36  (5.65–12.38) | <0.001 |

CI, confidence interval; n, number; OR, odds ratio; SE, standard error; Vx, vaccination.

## Table S2. Predictors of COVID-19 vaccination

|  | **Univariate analysis** | | | **Multivariate analysis** | | |
| --- | --- | --- | --- | --- | --- | --- |
|  | **OR** | **95% CI** | **p value** | **OR** | **95% CI** | **p value** |
| **Age ≥65 years** | 2.34 | 1.52–3.62 | <0.001 | 1.16 | 0.63–2.14 | 0.630 |
| **Australian** | 1.72 | 1.09–2.73 | 0.021 | 0.96 | 0.50–1.84 | 0.905 |
| **COVID-19: ADoH** | 11.65 | 7.22–18.79 | <0.001 | **4.36** | **2.39–7.97** | **<0.001** |
| **COVID-19: GP** | 7.14 | 4.57–11.2 | <0.001 | **4.57** | **2.48–8.42** | **<0.001** |
| **COVID-19: State health** | 15.17 | 6.88–33.45 | <0.001 | **8.62** | **3.24–22.94** | **<0.001** |
| **COVID-19: Other sources*** | 0.10 | 0.05–0.22 | <0.001 | **0.37** | **0.15–0.93** | **0.035** |
| **COVID-19: Social media** | 0.34 | 0.18–0.64 | <0.001 | 1.31 | 0.58–2.98 | 0.515 |
| **Exposure to fake news** | 0.61 | 0.38–0.97 | 0.037 | 0.68 | 0.35–1.33 | 0.260 |
| **Gender** | 1.87 | 1.24–2.82 | 0.003 | 1.69 | 0.95–3.01 | 0.076 |
| **HCP recommended** | 3.48 | 2.26–5.34 | <0.001 | **1.87** | **1.05–3.33** | **0.034** |
| **Medical condition** | 0.52 | 0.34–0.79 | 0.002 | 0.59 | 0.33–1.07 | 0.084 |
| **News: Religious leaders** | 0.26 | 0.09–0.74 | 0.012 | 0.36 | 0.08–1.54 | 0.168 |
| **News: Social media** | 0.56 | 0.38–0.84 | 0.005 | 0.64 | 0.35–1.18 | 0.152 |
| **News: Word of mouth** | 0.49 | 0.30–0.80 | 0.004 | 0.77 | 0.37–1.58 | 0.471 |

ADoH, Australian Department of Health; CI, confidence interval; GP, general practitioner; OR, odds ratio. *Such as, non-mainstream media/online sources, personal research/experiences, friends and family.

## Table S3. Vaccine confidence 3-factor scale item mean scores and factor loadings

| **8 items** | **Item Mean (SE)** | **Standardised factor loading**  **(SE)** | | |
| --- | --- | --- | --- | --- |
|  |  | **3-Factor Scale** | | |
|  |  | **Benefits** | **Harm** | **Trust** |
| COVID-19 vaccines are necessary to protect your own health. | 2.35  (0.07) | 0.91 (0.03) | - | - |
| COVID-19 vaccines do a good job in preventing the disease they are intended to prevent. | 2.20  (0.07) | 0.95 (0.02) | - | - |
| COVID-19 vaccines are safe. | 2.17  (0.07) | 0.94 (0.02) | - | - |
| If I do not vaccinate myself, I may get COVID-19 and cause others in the community to also get the disease. | 2.54  (0.07) | 0.87 (0.04) | - | - |
| I am concerned about getting COVID-19. | 2.56  (0.07) | - | 0.68 (0.09) | - |
| In general, the risk of serious side effects from COVID-19 vaccines (e.g. hospitalisation) is not higher than the risk of contracting the COVID-19 disease. | 2.49  (0.07) | - | 0.74 (0.09) | - |
| I have a good relationship with my general practitioner. | 3.70  (0.05) | - | - | 0.31 (0.07) |
| In general, the government and public health agencies in charge of COVID-19 vaccinations have my best interests in mind. | 2.02  (0.07) | - | - | 0.93 (0.13) |
| Cronbach alpha coefficient |  | 0.95 | 0.67 | 0.44 |

SE, standard error

# Supplementary data

## Two-part survey

## Factors that may influence vaccine confidence

1. **What gender do you identify as? [C]**
   1. Male
   2. Female
   3. Other
   4. Prefer not to say
2. **What is your age group? [C]**
   1. 18–24 years
   2. 25–34 years
   3. 35–44 years
   4. 45–54 years
   5. 55–64 years
   6. 65–74 years
   7. 75 years or older
   8. Prefer not to say
3. **Please specify your ancestry (tick up to two only) [C]**
   1. Australian
   2. Aboriginal
   3. Torres Strait Islander
   4. Chinese
   5. English
   6. Filipino
   7. German
   8. Irish
   9. Indian
   10. Italian
   11. Scottish
   12. Other ancestry 1 (please specify below)
       1. _________________________________
   13. Other ancestry 2 (please specify below)
       1. _________________________________
   14. Prefer not to say
4. **What is your religion? [C]**
   1. Christianity/Catholicism
   2. Judaism
   3. Islam
   4. Buddhism
   5. Hinduism
   6. No religion
   7. Other (please specify below)
      1. _________________________________
   8. Prefer not to say
5. **What is the highest level of education you have completed? [C]**
   1. Less than high school
   2. High school
   3. Bachelor’s degree
   4. Master’s degree
   5. PhD or higher
   6. Trade school
   7. Prefer not to say
6. **What is your weekly household income? [C]**
   1. $1–500 per week
   2. $501–1000 per week
   3. $1001–1500 per week
   4. $1501–2000 per week
   5. $2001–2500 per week
   6. >$2500 per week
   7. Prefer not to say
7. **Do you have any of the following conditions? (tick all that apply) [C]**
   1. None
   2. Asthma
   3. Cancer
   4. Current smoker
   5. Chronic kidney disease
   6. Chronic obstructive pulmonary disorder
   7. Cystic fibrosis
   8. Diabetes
   9. Down syndrome
   10. Heart disease (e.g. heart failure, coronary artery disease, cardiomyopathies)
   11. HIV
   12. Hypertension
   13. Immunocompromised or immunosuppressed
   14. Liver disease
   15. Neurological conditions (e.g. Alzheimer’s disease, dementia)
   16. Obesity
   17. Other lung diseases (e.g. interstitial lung disease, pulmonary fibrosis, pulmonary hypertension)
   18. Previous stroke
   19. Sickle cell disease
   20. Solid organ or blood stem cell transplantation
   21. Substance use disorders
   22. Thalassemia
   23. Prefer not to say
8. **Has a doctor or other healthcare professional ever recommended that you get a COVID-19 vaccine? [S]**
   1. No
   2. Yes
9. **Do you identify yourself as a healthcare professional, researcher or working in a job related to healthcare? [C]**
   1. No
   2. Yes
10. **Where do you receive your news from? (tick all that apply) [S]**
    1. Community/religious leaders
    2. Online news articles
    3. Print news (e.g. newspapers, magazines)
    4. Radio
    5. Social media
    6. Television news broadcast
    7. Word of mouth
    8. Other (please specify below)
       1. _________________________________
11. **Have you seen or heard any information about COVID-19 vaccines (e.g., on the news, on social media, or from friends and family) that you could not determine were true or false? [S]**
    1. No
    2. Yes
12. **Select your top 3 most trusted sources of information about COVID-19 vaccines: [S]**
    1. Australian Department of Health
    2. Employer
    3. General practitioner
    4. Hospital system websites (e.g. Blacktown & Mount Druitt Hospital website)
    5. News sources (e.g. television, internet, radio)
    6. Nurses
    7. NSW Health
    8. Online publishers of medical information (e.g. WebMD or Mayo Clinic)
    9. Pharmacists
    10. Professional organisation(s)
    11. Religious Leader(s)
    12. Social media (e.g. Facebook, Twitter, Instagram, WhatsApp, LinkedIn, Tik-Tok)
    13. Therapeutic Goods Administration
    14. Union leader(s)
    15. Other (please specify below)
        1. _________________________________
13. **Have you or anyone in your family or friends had COVID-19?** **[S]**
    1. No
    2. Yes
    3. Prefer not to say
14. **Have you received a vaccination for COVID-19?**
    1. No
    2. Yes
15. **What would motivate/motivated you to get vaccinated? (tick all that apply**) **[M]**
    1. Protect my health
    2. Protect the health of family/friends
    3. Protect the health of colleagues
    4. Protect the health of my community
    5. To get back to work/school
    6. To resume social activities
    7. To resume travel
    8. Because others encouraged me to get vaccinated (please specify below)
       1. _________________________________
    9. Other (please specify below)
       1. _________________________________
    10. Not sure
16. **In terms of accessibility, how difficult is it/was it for your chosen COVID-19 vaccine? [P]**
    1. Not at all difficult
    2. A little difficult
    3. Neutral
    4. Somewhat difficult
    5. Very difficult

## Vaccine Confidence Scale

**How much do you agree or disagree with the following statements?**

1. ***COVID-19 vaccines are necessary to protect your own health* [B]**
   1. Strongly disagree
   2. Disagree
   3. Neither agree nor disagree
   4. Agree
   5. Strongly Agree
2. ***COVID-19 vaccines do a good job in preventing the disease they are intended to prevent* [B]**
3. Strongly disagree
4. Disagree
5. Neither agree nor disagree
6. Agree
7. Strongly Agree
8. ***COVID-19 vaccines are safe* [B]**
   1. Strongly disagree
   2. Disagree
   3. Neither agree nor disagree
   4. Agree
   5. Strongly Agree
9. ***If I do not vaccinate myself, I may get COVID-19 and cause others in the community to also get the disease* [B]**
   1. Strongly disagree
   2. Disagree
   3. Neither agree nor disagree
   4. Agree
   5. Strongly Agree
10. ***I am concerned about getting COVID-19* [H]**
    1. Strongly disagree
    2. Disagree
    3. Neither agree nor disagree
    4. Agree
    5. Strongly Agree
11. ***The risk of serious side effects from COVID-19 vaccines (e.g. hospitalisation) is not higher than the risk of contracting the COVID-19 disease* [H]**
    1. Strongly disagree
    2. Disagree
    3. Neither agree nor disagree
    4. Agree
    5. Strongly Agree
12. ***I have a good relationship with my general practitioner* [T]**
    1. Strongly disagree
    2. Disagree
    3. Neither agree nor disagree
    4. Agree
    5. Strongly Agree
13. ***In general, the government and public health agencies in charge of COVID-19 vaccinations have my best interests in mind* [T]**
    1. Strongly disagree
    2. Disagree
    3. Neither agree nor disagree
    4. Agree
    5. Strongly Agree

KEY

C: Conditional Influences; S: Social Influences; M: Motivational Influences; P: Practical Influences; B: Benefits; H: Harms; T: Trust.
